# “She gives it to her child who doesn’t even talk”: a qualitative exploration of alcohol and drug use among primary school-age children in Uganda

**DOI:** 10.1186/s12889-023-17016-5

**Published:** 2023-10-27

**Authors:** Joyce Sserunjogi Nalugya, Vilde Skylstad, Juliet N Babirye, Andrew Sentoogo Ssemata, Grace Ndeezi, Paul Bangirana, Ingunn M. S. Engebretsen, Noeline Nakasujja

**Affiliations:** 1 Department of Psychiatry, Mulago National Referral and Teaching Hospital, Ministry of Health, Kampala, Uganda; 2 Department of Psychiatry, School of Medicine, College of Health Sciences, Makerere University, Kampala, Uganda; 3 Centre for International Health, Department of Global Public Health and Primary Care, Faculty of Medicine, University of Bergen, Bergen, Norway; 4 School of Public Health, Makerere University College of Health Sciences, Kampala, Uganda; 5 Department of Global Health and Development, London School of Hygiene and Tropical Medicine, London, UK; 6 Department of Paediatrics and Child Health, School of Medicine, College of Health Sciences, Makerere University, Kampala, Uganda

**Keywords:** Primary school-age children, Alcohol use, Drug use, Perceptions, Qualitative research, Uganda

## Abstract

**Background:**

There is little research on alcohol and other drugs (AOD) use by school-age children in low-resource settings like Uganda. Including the voices of children in research can inform prevention and early intervention efforts for those at risk of AOD use. The aim of this study was to understand the perspectives of children aged 6 to 13 years regarding AOD in Uganda.

**Methods:**

This qualitative study was conducted in Mbale district, Uganda from February to March 2020. Eight focus group discussions (FGDs) were conducted with 56 primary school-age children, stratified by age (6–9 and 10–13 years), sex (male and female), and school status (in school and out of school). All FGDs were conducted in either Lumasaaba or Luganda. The FGDs were audio-recorded, transcribed verbatim, and translated into English. Data were coded, and overarching themes were identified using thematic framework analysis.

**Results:**

Two themes identified were (1) Children’s perceptions and experiences with AODs. The participants understood alcohol by its consistency, colour, odour, and by brand/logo. They described the types and quantities of AOD consumed by school-age children, brewing processes for homemade alcoholic drinks, and short and long-term consequences of the use of alcohol. (2) Contributing factors to childhood drinking included: Stress relief for children who experienced multiple adversities (orphaned, poverty-stricken, and hailing from broken homes), fitting in with friends, influence from families, and media exposure that made alcohol look cool. Children would start drinking at an early age) or were given alcohol by their parents, sometimes before they could start talking. In the community, alcohol and other drugs were cheap and available and children could drink from anywhere, including in the classroom.

**Conclusions:**

Children eligible for primary education in Uganda can easily access and use AOD. Several factors were identified as contributing to alcohol and other drug use among children, including availability and accessibility, advertising, lack of parental awareness and supervision, peer influence, adverse childhood experiences, socioeconomic factors, and cultural norms. There is a need for multi-sectoral action for awareness of childhood AOD use and deliberate consideration of children in the planning, design, and implementation of research, policies, and programs for prevention and early intervention.

## Introduction

Recent research in Uganda highlights a concerning trend of alcohol use among young children between the ages of 5 and 8 years [1]. Adults interviewed in previous studies have revealed that many children are exposed to alcohol and other drugs within their families due to poverty and lack of resources [2]. This observation sparked further investigation into the underlying factors that contribute to alcohol and other drugs (AOD) use by children in Uganda, with a focus on giving voice to the children themselves.

From a developmental perspective, children’s drinking behaviors are shaped by the social contexts in which they live [3]. The bioecological theory of human development, proposed by Bronfenbrenner [4], emphasizes the significance of proximal processes—interactions within immediate and more remote environments—consistently occurring over time. In the context of childhood alcohol use, this theory highlights that children acquire attitudes and behaviors related to alcohol from their families, schools, and neighborhoods. However, the expression of these behaviors depends on how children perceive alcohol in their immediate surroundings and the ongoing processes in their broader environments.

Harmful alcohol use among older adolescents and adults (15 years and above), is a significant health concern, responsible for over 3 million deaths annually and contributing to over 200 diseases and health conditions worldwide [5]. In 2016, alcohol consumption was responsible for 3.8% of female deaths and 12.2% of male deaths in the age group 15 to 49 years globally [6]. This public health issue not only affects adults but also raises concerns about its indirect impact on children. The behaviors and attitudes surrounding alcohol use in adult populations can profoundly influence the perceptions and experiences of children within their immediate environments.

Research indicates that the use of AOD use is a noteworthy concern among children and adolescents in low- and middle-income countries (LMICs), especially in regions such as sub-Saharan Africa and Southeast Asia. Studies in these areas have established a connection between adolescent exposure to household alcohol misuse and adverse outcomes, including mental health issues, problem behaviors, and suicidality [7, 8].

Adolescents in sub-Saharan Africa have been found to have high rates of AOD use, with evidence suggesting that they are starting to drink at increasingly younger ages [9, 10]. However, despite this notable trend among adolescents, there is a noticeable gap in the available data pertaining to alcohol consumption among school-age children [11, 12]. This is particularly concerning in Uganda, where alcohol is widely available and homemade varieties are largely unregulated. A 2004 survey found that Uganda was among the highest alcohol-consuming countries, with per capita annual consumption reaching 19.5 L among individuals aged 15 years and older [13, 14].

Alcohol is a psychoactive substance that is widely used, and research has shown that it can serve as a gateway to other drug use among adolescents [15]. Psychoactive substances like alcohol affect the central nervous system and alter a person’s consciousness, thinking, feelings, perception, and behaviour in response to their surroundings [16]. As such, exposure to alcohol during any stage of childhood and adolescence can have negative effects on the brain both in the short and long term [17, 18]. In the short term, alcohol use can increase a child’s vulnerability to violence, accidents, injuries, and death [19]. Adolescents who consume alcohol are more likely to engage in risky behaviours including driving under the influence of alcohol and unsafe sex [20]. They may also experience alcohol overdose, which can be life threatening. In the long term, studies have shown that early onset of alcohol use can have serious consequences for physical and mental health. Individuals who start drinking alcohol before the age 13 years, are likely to develop alcohol use disorders and experience other problems later in life [21, 22].

Alcohol consumption by children can have far reaching consequences that extend beyond their physical and mental health. For example, alcohol can interfere with the Childrens’ academic achievement by impairing cognitive and executive functioning, leading to poor school performance and increased risk of school-drop out [23]. Alcohol use can also increase the risk of exposure to infections, including HIV/AIDs, and increase the adolescent’s vulnerability to violence and victimization, which can impact not only the individual but also their families and communities [24].

While alcohol policies aimed at discouraging underage drinking are in place in numerous countries, it continues to pose a significant worldwide public health challenge [25]. Alcoholic brands continue to target young people through advertising and marketing including those implemented on social media platforms [26, 27].

Given the complex and interconnected nature of these issues, preventing underage drinking requires a multi-faceted approach that addresses the underlying factors that contribute to alcohol use among children. Despite the growing concern about AOD use among school-age children in low-resource settings such as Uganda, there remains limited research on this issue. In particular, children’s voices are absent yet capturing the perspectives of children themselves can inform effective prevention and early intervention efforts for those at risk of alcohol and other drug use.

This study set out to address the research gap concerning AOD use among school-age children in underserved contexts such as Uganda. By incorporating the viewpoints of children aged 6 to 13 years, the target age for primary school, our research could enhance the foundation for effective prevention and early intervention strategies for children at risk of AOD use. Specifically, the aim of this study was to understand the perspectives of children aged 6 to 13 years regarding AOD in Mbale district, eastern Uganda.

## Methods

### Design and setting

We employed an exploratory qualitative study design [28], using focus group discussions (FGDs) to investigate children’s views on AOD use by children [29]. This design allowed researchers to evaluate the participants’ perspectives on and experiences with AOD use by children in their environments. It was considered most appropriate to allow the participants to provide their own narratives on AOD [30].

The study was conducted in Mbale district, eastern Uganda, from February to March 2020 just before school closure due to the COVID-19 pandemic. The district hosted the TREAT C-AUD project that investigated the magnitude of alcohol and other drug use among 6–13-year-old children in the health, school and community contexts [31]. At the time of the study Mbale city was a municipal council consisting of three divisions. The city lies at the western foot of mount Elgon about 225 km by road to the northeast of Kampala, Uganda’s capital.

### Participants

Participants were primary school-age children in or out of school purposively sampled to maximize representativeness [32]. The inclusion criteria were: (i) the child had to be staying under adult supervision (with parents or adult care-givers avoiding street children or children in child-headed household), (ii) Had to be in 6 to 13 years age range, (iii) had lived in Mbale district for one or more years, and (iv) the parents had to consent for the child to participate in the study. Children who were ill were excluded. Participants were recruited from eight cells (villages) within the three divisions of Mbale city. The researcher sought the support of local administrative leaders at village level to access the households. Verbal informed consent was obtained from parents and assent from the children and the parents contacts were kept for later appointments. Out of the 70 parents approached, 56 consented to their children’s participation in the study. Some of the parents who did not consent had fears of their children being exposed to bad practices of using alcohol, yet others gave no reasons.

Participants were allocated to groups according to age, gender, and school status. No children from the same village were in the same group to minimize the impact of local social interactions during the discussions [29]. Eight focus groups with 6–9 participants in each were stratified by age (6–9 years and 10–13 years), gender (boys and girls), and school attendance status (in school and out of school). The age, sex, school status, and size of groups are given in Table 1.

**Table 1:** Focus group composition by age, sex, school status (n = 56)

Focus Group Discussion	Category	Number of participants
1	Boys 6 to 9 years in school	7
2	Girls 6 to 9 years in school	8
3	Boys 6 to 9 years not in school	6
4	Girls 6 to 9 years not in school	6
5	Boys 10 to 13 years in school	9
6	Girls 10 to 13 years in school	7
7	Boys 10 to 13 years not in school	7
8	Girls 10 to 13 years not in school	6

### Procedure

One day prior to the FGD, a member of the research team (RN), contacted the parents of the potential child participants by phone or home visit. Parents were asked to come with their children to a safe, quiet place which was identified within the community. On the day of the FGD, informed written consent from the parents and assent from the participants were obtained by JSN and RN. The parents were allocated a safe place to wait for their children until the end of the FGD. We ensured that parents were informed about these provisions beforehand, and they willingly agreed to participate under these circumstances. Participants were reassured of confidentiality throughout the FGDs and later data handling. We tried as much as possible to observe the children’s rights to privacy during the study processes. The information shared in the group discussions was not shared with parents or other elders and the participant’s names were protected.

### Data collection and instruments

Data was collected using a focus group discussion guide which was formulated by the research team based on the socio-ecological model, as a theoretical framework [33]. We used open-ended questions focused on individual, family, peer, school and community factors that could be related to childhood substance use. The guide was developed in English, and later translated to the local languages (Lumasaaba and Luganda). The translated FGD guide was back translated and thereafter pre-tested among children of the same age group in Mbale district outside Mbale city. The guide was then revised by removing some duplicates and simplifying difficult words. Table 2 shows the five topics that guided all the eight focus group discussions.

All the FGDs were conducted by the first author (JSN), an experienced child psychiatrist, as facilitator assisted by two experienced social workers (RN) and (WB) who were conversant with the Lumasaaba, Luganda and English languages. During the FGDs, study numbers were used to refer to participants which were allocated to them after the recruitment. The participants were encouraged to interact freely, and to use their preferred languages (Lumasaaba, Luganda or English). They were allowed to have short breaks and each participant was given a soft drink and a snack. Participants were allowed to ask any questions before closure of the discussions and each FGD lasted approximately 90 to 100 minutes. After the study, JSN and WB conducted site visits to find out the availability and percentage of alcohol by volume (ABV) contained in the alcoholic beverages that the participants mentioned in the study. Confirmation of the availability of alcoholic beverages and substances was conducted as part of an observation phase that followed the initial data collection process. This phase aimed to verify the accuracy and validity of the information provided by the participants during the interviews. It did not constitute a separate follow-up study. We conducted the observation phase openly, and the businesses where alcoholic beverages were available were fully aware of our presence and intentions (some allowed us to take photos). Verbal consent for our presence and observation was obtained from the business owners or managers.”

**Table 2:** Focus Group Discussion Guide Topics in English, Lumasaaba and Luganda

English	Lumasaaba	Luganda
What is your understanding of alcohol?	Shiina iwowe shesi utegela nni nguuli?	Kiki ky’otegeera oba kyomanyi ku mwenge?
How common do you think it is for children to drink alcohol and use other drugs?	Ubaasa shili shetsindaalo abaana khunywa inguuli nni khurambisa ebindu bindi byekhumesa?	Olowooza kitera okubaawo abaana abato okunywa omwenge n’okukozesa ebiragalalagala?
How do children in this community get the alcohol and other drugs?	Abaana mushisinza shino bafuna baryeena inguuli nni bimeesa bindi?	Abaana mu kitundu kino bafuna batya omwenge n’ebiragalalagala ?
What kinds of problems do you see in children who use alcohol or other drugs in your community?	Bi’angafu shiina byesi uboona mu baana banywa inguuli nni khurambisa ebimesa bindi mushisinza shenywe	Bizibu bya ngeri ki by’olaba mu baana abakozesa omwenge oba ebiragalalagala mu kitundu kyo oba eyo ewamwe gyobeera?
What happens to the children who drink alcohol or use other drugs?	Shiina shikholikha khu baana banywa inguuli nni khurambisa ebimesa bindi?	Biki ebituuka ku baana abanywa omwenge oba abakozesa ebiragalalagala?

### Ethics

Ethical approval was obtained from Makerere University School of Medicine Higher degrees Research Ethics Committee (SOMHDREC) Ref. 2018-095), the Uganda National Council for Science and Technology (UNCST) Ref. SS5103 and the Norwegian Research Council #50,146. Permission was also obtained from the Chief Administrative Officer (CAO) Mbale district administration and the study area local council chairpersons prior to data collection. Both verbal and written informed consent was obtained from parents and assent was obtained from all the child participants. Verbal explanations were utilized to obtain participation agreement from both parents and children. This enabled us to accommodate parents with limited literacy while ensuring comprehension for child participants, regardless of their developmental stages. For parents who faced challenges with reading and writing, consent was obtained through the use of thumbprints as an alternative form of endorsement. Parents were compensated for the time spent and given a transport refund.

In anticipation of potential child protection concerns, a comprehensive risk mitigation plan was developed as an integral part of the study protocol. It was designed to ensure the safety and well-being of child participants throughout the study duration. The plan, developed during the initial stages of study planning prior to data collection, employed a multi-layered approach to safeguard participants. The key components of the plan included sensitizing the research team to address child protection issues, establishing an informed consent process that prioritized participant understanding and concerns, implementing confidentiality measures to safeguard identities, creating a safe environment where participants could freely voice any concerns and designating dedicated focal persons (RN and JSN) to handle child protection matters, including referrals to Mbale regional referral hospital and other agencies when needed.

### Data analysis

The FGDs were audio recorded, transcribed verbatim and translated to English. Data was analysed using thematic framework analysis [34]. Two authors (JSN and ASS) read and reread the transcripts until they were familiar with the data. The researchers reviewed the field notes generated during the Focus Group Discussions (FGDs). The notes were subsequently combined with the corresponding audio recordings to capture participants’ perspectives, enhancing transcription clarity. Key ideas were recorded, and initial codes and categories were generated. The analysis was conducted in two phases subsequent to the completion of reading all the FGDs. In the first phase, two FGD transcripts were coded independently by the two authors and an initial coding framework were developed and agreed upon. Thereafter, the two authors together with the research team reached a consensus about the identified codes and categories and discussed potential themes. Five transcripts were coded and themes were identified and compared to ensure consistency. The codes generated were used to develop a refined analytical framework and codebook. In the second phase, the developed codebook was used to examine and manually index the remaining transcripts through an iterative process, adding new codes which reflected the agreed themes and sub-themes as they emerged. Thereafter, overarching themes and sub-themes were identified from patterns in the framework matrix and iteratively through discussion of the data. In this paper we present the overall analysis of data from the FGDs. A number of quotes were identified from the transcripts during the initial analysis process of this study. The quotes used in the results section were selected basing on the representativeness of a specific perspective or being illustrative of a recurring pattern to provide a representation of the participants’ perspectives.

To interpret the results of the FGDs, it should be noted that the legal age to purchase and consume alcohol in Uganda at the time of data collection was 18 years and the children were informed that alcohol use did not include drinking a few sips for religious or cultural purposes.

## Results

In this section, we present the rich and insightful perspectives shared by children aged 6 to 13 years regarding alcohol and other drugs (AOD) in Uganda, as revealed through our qualitative exploration. These findings provide a comprehensive understanding of their views and experiences, shedding light on the social ecological factors related to AOD use by children in Uganda.

### Thematic findings

We present children’s perceptions and experiences with alcohol and other substances, how children access alcohol and drugs and factors contributing to why children drink. The findings were informed by the children’s own experiences and how they had observed other children in their environments regarding alcohol and other drug use. An overview of themes and sub-themes is given in Table 3.

**Table 3:** Themes and sub-themes from the FGDs

Themes	Sub-themes
Children’s perceptions and experiences with alcohol and other drugs	Knowledge about alcohol Types and quantities of alcohol consumed Knowledge of other drugs Consequences of alcohol and other drug use
Contributing factors to childhood alcohol and other drug use	Children experiencing multiple adversities Care providers and peer influences Alcohol was easily accessible and children could drink anywhere Media made alcohol look cool

### Children’s perceptions and experiences with alcohol and other substances

The participants in the FGDs were knowledgeable about alcohol and other drugs, how alcohol was locally manufactured, and the types available in their communities. They shared experiences with the quantities consumed by children and the consequences of AOD on those who used them.

#### Knowledge about alcohol

‘In nearly all the FGDs, participants discussed about their awareness of alcohol, exemplifying their familiarity with a variety of alcoholic beverages and their capacity to distinguish between spirits and beers. They precisely described various types including homemade alcoholic beverages and those sold in bars and shops. For instance, they described spirits, commonly known as ‘waragi’ or ‘Enguli’ (local dialect). They described spirits as looking like water in consistence and colour and could only be differentiated from water by looking at the packaging materials or by its smell. One participant in an FGD of boys in school described the different types of spirits commonly sold on the market:

*“Cock gin, Ambiance waragi, Reading, Coffee, Empire, Simba, Digital gin, Uganda waragi, Ex-five, Empire, Uganda premium gin. These are all sold a lot in ‘kaveera’ [plastic pack]”* (Boys’ FGD, 10–13 years in school).

Beer was also mentioned in all FDGs as one type of alcoholic drinks in the communities. The children explained that the beers were usually sold in dark coloured bottles with different types of branding. Children expressed ease of identifying the appearance of different brands of alcoholic beverages that children in their communities would drink.

*“Beer is another type of alcohol. Beer is the one in the big dark bottles like Long Nile, Nile special, Club Pilsner, Eagle lager [beer brands].”* (Girls’ FGD, 6–9 years out of school).

The locally made alcoholic beverages drunk by children were clearly described according to the different varieties based on the different types of food used to make the alcoholic drinks. These were mainly from ripe bananas (matooke), maize and millet, but also other types were described.

*“Others drink malwa [an alcoholic drink made from fermented millet mixed with maize product] and others drink Mwenge bigele [fermented bananas] or Kitoko sold in kaveera [plastic pack]. It is like water but it tastes sour and when you drink it, it smells from the mouth.”* (Boys’ FGD, 10–13 years in school).

#### Types and quantities of alcohol consumed

The participants had a detailed and practical understanding about how alcohol was brewed, stored and sold.

*“Kitoko (local brew), it is white. You go with a bottle and they (the merchants) pour for you in the bottle.”* (Girls’ FGD, 10–13 years in school).

The locally made alcoholic beverages would be taken in plastic cups or other containers like mineral water bottles.

*“Someone can drink 4–10 cups every day. When he tastes and finds that it is sweet then he says that I will be buying this type. Every day he buys when he tastes and its sweet.”* (Boys’ FGD, 6–9 years in school).

The factory-made types would be described as packets, sachets, small bottles (100–250 mls) and larger/longer bottles of beer (500 mls).

*“Some drink one, others three and others six small bottles of alcohol (spirit). Two people can buy their pack (a box containing 6 to 12 small bottles of alcohol [in the supermarket]) and they sit under the tree and they drink it all.”* (Boys’ FGD, 10–13 years out of school).

Participants in the 10–13-year age group reported children consuming larger quantities of alcohol, and a higher frequency of intake, compared to the younger participants. Some of the older children explained that they knew of peers who drank several units of beer and spirits, sometimes even daily.

*“Yes, there is a child who can drink 2 bottles of beer. He is slightly older than this one (pointing to a younger child next to him). He drinks from Kikindu (a nearby village).” (*Boys’ FGD, 10–13 years in school).

#### Knowledge of other Drugs

In addition to alcohol, participants discussed the existence of other intoxicating substances in the communities where they lived, the access and use by children. Some drugs such as marijuana could be purchased or accessed from the gardens and nearby retail shops or markets. Marijuana was available in various forms such as fresh leaves (for chewing) and dry leaves for smoking. Other drugs were: ‘Gum’, aviation fuel, tobacco, ‘mairungi’ (khat/mirrah), ‘kuber’ (narcotic drug of Indian origin), shoe polish and nail vanish. However, alcohol was viewed as being much cheaper compared to leafy drugs that were chewed, such as ‘mairungi’ (khat).

*“Alcohol and ‘Gum’ are easy to get but ‘Mairungi’ is not easy because it is expensive and it needs to be accompanied by big G [chewing gum that hides the bitter flavour of Khat]. Where there is big G, just know that there is ‘Mairungi’ [khat]. Big G is sold at 200 Ushs. (USD = 0.053), Mairungi is sold as a bundle of leaves at 500 Ushs. (0.14 USD)”* (Girls’ FGD, 10–13 years in school).

The children also reported that there was a tendency of some children to smoke marijuana in groups.

*“You find when they have put it (marijuana) on the table, and they are seated in a group when they are inhaling the smoke from the nose. They roll the leaves in a piece of paper, light it and they puff. They tell you to inhale, that you will feel good. After this you can feel dizzy”.* (Boys’ FGD, 10 to 13 years in school)

Additionally, participants in the 10-to-13-year age and out of school groups described use of other forms of drugs such as injectables which children could purchase and inject themselves.

*“There are some drugs which others just inject and they sleep. Others buy and inject here like this [demonstrates on cubital fossa on the forearm]. They buy it from town. It has a bottle that looks like those for hospital medicines. They just insert the needle and syringe in the bottle and then they inject into their arm and their eyes become red.”* (Boys’ FGD, 10–13 years out of school).

Although the majority of the children knew about alcohol, children in school seemed to describe fewer types of substances than the children who were out of school.

#### Consequences of alcohol and other drug use

Many participants discussed the negative aspects of childhood AOD use. These included the unpleasant body smell and breath among those who drank alcohol. The youngest children confirmed this understanding. In addition, they described alcohol as a drink that affects the brain, and would make people drunk, and forgetful, and it would cause sleepiness, dizziness, headache, and make people prone to accidents and death.

*“Alcohol smells and when you drink it you smell. You smell in the mouth and everyone realizes that you have drunk alcohol*”. (Boys’ FGD, 6–9 years out of school)

*“…When they drink alcohol, they forget what they are supposed to do because their brains don’t work well.”* (Girls’ FGD, 10-13years in school).

The participants described a fear of being harmed by children who drank alcohol or used other psychoactive substances. These fears included risk of being raped and exposure to sexually transmitted illnesses and physical injury. The participants explained that this could happen anywhere at home, school, on the way or other places.

*“Those who take alcohol commit crimes. They can find you walking at night and they rape you. When they find their friend in the forest looking for firewood, they attack them and rape them.”* (Girls’ FGD, 6 to 9 years in school).

The FGD of the youngest participants attributed risky behaviours to AOD use, such as fighting, lying and stealing. Drunk children could place their own lives and the lives of others in danger. They discussed circumstances where mothers would be arrested by police after their children drank alcohol, fell and sustained injuries. Additionally, children who used AOD could become verbally and physically violent towards other children, parents and teachers who commented on their drinking. The participants explained that this could make it difficult to advise and help those who drank alcohol.

*“…You see him hiding behind the toilet (at school) while drinking alcohol, afterwards he becomes rude. When you tell him that alcohol will affect you negatively, he just becomes rude to you. Then you just leave him. Because when you advise him, he beats you and he says ‘Leave me to drink, it is not yours. It is me who bought’”.* (Boys’ FGD, 6 to 9 years in school)

The consequence of such behaviour usually received corporal punishment from their parents or teachers.

*“Children can drink alcohol and they come back home when they are rude. When their father tries to advise them, they get annoyed. When the father tries to beat the child (punishment) then the child feels proud, then he begins to fight/beat him back.”* (Girls’ FGD, 10 to 13 years in school).

Those who use marijuana and other drugs were described as having red eyes, black teeth and behaving like ‘mad’ people (i.e., with severe mental illness). The participants also discussed academic challenges with poor performance and possibility of ending up in prison. Despite the negative consequences of alcohol and other drug use, the majority of participants discussed that children could easily drink a lot of alcohol and some used other drugs.

### Contributing factors to childhood alcohol and other drug use

The reasons for alcohol and other drug use by children included individual, family and community factors. Factors at individual level included stress from multiple adversities including orphanhood, poverty, broken homes and school-related situations. They described the easy access and use in families that drunk alcohol facilitated by siblings and parents. They also described peer influence and community factors such as availability of cheaper brands, the selling of AOD to children, and media promotions as contributors to childhood drinking.

#### Children experiencing multiple adversities

Some participants described how children could experience extreme and multiple adversities such as separation from or death of parents, poverty that resulted in lack of resources to cover basic needs like food, and verbal, emotional and physical violence from primary caregivers. Such situations would cause severe stress and anxiety that could result in using alcohol and other substances which were perceived to give a ‘peace of mind’.

*“Others may be orphans when their mothers died and they stay with the step-mother, and their step-mother mistreats them and they leave home and go to the streets and start drinking alcohol.”* (Girls’ FGD, 10–13 years in school).

School related stress was important, and included pressure to perform, excessive homework in addition to beating by the teachers.

#### Care providers and peer influences

The participants delineated that some children drank alcohol because they had learned it from their family environment, and they were more likely to drink at an early age. The age of initiation of alcohol intake varied and many were exposed quite early.

*“Three or four years and children are drinking. Others drink alcohol at five years and others, like me, at six years.”* (Boys’ FGD, 6–9 years in school).

Some parents encouraged their children to drink by giving them to taste or accepting occasional drinking at parties or ceremonies.

*“Sometimes fathers tell children to taste alcohol. They get from the bar or at parties or ceremonies or in the shop or sometimes fathers bring for them what to drink. Sometimes the father can be bathing and the children ask where the bottle is (child stealing father’s alcoholic drink)”.* (Boys’ FGD, 10–13 years in school)

Younger children, however, explained that their parents would permit them to drink, but limit the quantities consumed.

*“A parent can allow you to take one cup of Malwa (local brew)). The mother says you are still young, let me give you little alcohol”.* (Boys’ FGD, 6 to 9-years in school)

Family members were sometimes facilitators to children’s alcohol intake by providing or encouraging alcohol.

*“There is a child I know about, when his father leaves an alcohol bottle on the table he picks it and drinks. Sometimes the fathers themselves tell their children to take the alcohol”* (Boys’ FGD, 10–13 years in school).

Childhood drinking was partly explained by poverty related challenges, where lack of food could be mitigated by giving the child alcohol to suppress hunger.

*“The children come to their mother, and they see her drinking alcohol, when the mother realizes that the children are looking at her, she gives them alcohol too when they are hungry”* (Boys’ FGD, 6 to 9 years, in school).

Parents could also negotiate difficult situations resorting to alcohol. The participants discussed parents who gave their very young children alcohol to help them sleep.

*“There is a woman next to where I stay who gives her children alcohol called V&A [sherry with 20% ABV]. She gives it to her child who doesn’t even talk like when she wants the child to sleep.”* (Boys’ FGD, 10–13 years out of school).

Participants narrated that although alcohol consumption was an unacceptable practice for children, children consume alcohol because of peer influence. They elucidated that children would drink alcohol with their peers and, in hiding before throwing the bottles away. Also, the participants explained that the children who drink more alcohol were considered more energetic and popular. According to the discussions, there were children who drank alcohol to become popular while some children drank alcohol to be brave, since it was not for the weak and faint hearted.

*“Some children drink because they want to be recognized among those who drink. They want to be called energetic because they say it gives them energy and when he sees those famous people drinking, he also admires to drink.”* (Boys’ FGD, 10–13 years in school).

#### Alcohol was easily accessible and children could drink anywhere

Participants discussed in detail the extensive availability and accessibility of alcohol to children regardless of age, sex or whether children attended schools or not. The participants explained that alcohol was cheap, and affordable even for the primary school - age children. Some alcoholic drinks were packaged in small bottles and a child did not need a lot of money to purchase them. Alcohol could easily be obtained for as low as 200–500 Uganda Shillings (0.05–0.15 USD). Some payment in cash or in kind was required, children would work, steal, or deceive adults in order to get money to buy it.

*“They buy it from the bars or clubs. If they do not have money they go and fetch firewood for the owner then they give them alcohol in return for the work”* (Girls’ FGD, 10–13 years out of school).

Other places where children easily accessed alcohol included homes, school, neighbourhoods, shops, cinema halls, parties, and even supermarkets. The cost for the alcohol was said to be higher at the supermarkets.

*“Another alcohol is in the long bottles (beer) when it has a mark (branded) and they sell it Ushs.5000/= (USD = 1.32) in the supermarket. Yes, young children buy alcohol for Ushs. 5000/= from the supermarket and they drink and finish the contents of the bottle.”.* (Boys’ FGD, 6 to 9 years out of school).

They discussed that when a child is given money to buy other items and they remain with spare change or balance, they could decide to buy and drink alcohol. Also, children from wealthy families with money at their disposal ended up spending it on alcohol.

*“Some children request their parents for money to buy books and they instead buy “waragi” (local spirit).”* (Boys’ FGD, 6- to 9-years out of school).

They described the mixing of alcohol with other soft drinks like soda or other food stuffs.

*“Others drink alcohol that is like soda but when it is bitter. Others buy ‘waragi’ (strong homemade spirit) and mix with soda. There are others who mix alcohol with avocado and they put in food and eat.”*. (Boys’ FGD, 6 to 9 years out of school)

Children who drank from school devised means of hiding or using water bottles to disguise spirits to escape being noticed and punished.

*“He can get a small mineral water bottle and pour alcohol in it. He can lie that someone has sent me (to bring it for him/her) and yet he is going to drink from school. He then drinks from class even when the teacher is teaching. He bends and hides under the desk; he sips and puts the bottle back in the bag. He can’t listen to what the teacher is teaching.”* (Boys’ FGD, 6–9 years in school).

#### The media made alcohol look ‘cool’

In some instances, alcohol was accessed at no cost especially during the time of public exhibitions and street promotions of alcoholic beverages which were common and attended by people of all ages, including children. Children got free drinks from promotional vehicles to have a feel of how the new product on the market tasted. The promoters aimed to make people think that the new product is superior to the others already on the market.

*“Children can get alcohol from the promotional trucks and they give it out free (at no cost). For example, the advertising truck can be announcing the coming of [name of famous music artiste] to perform and the promoter say, ‘what do we drink!!!’ while they throw Cock gin [brand of gin] off the truck for people and they put a song of the musician and the crowds reply ‘Cock gin’. They throw the tot-packs and people pick them, young and old drink the free alcohol. Children like to follow those trucks everywhere.”* (Boys’ FGD 10–13 years out of school).

The participants discussed that children drank for the first time at school events such as sports-day or other ceremonies.

*“Others when it is ‘sports-day’, they come with those things (alcoholic beverages) at school they start drinking and even give to their friends those who don’t know alcohol”* (Girls’ FGD, 10–13 years in school).

In addition to accessibility of alcohol to children from business promotions, advertising also contributed to alcohol use by children. Whenever there was a new alcohol product on the market, the manufacturers associated it with a celebrity or famous artist and moved to the communities to promote their products. Anyone attending would get a sample of the displayed product, regardless of age. The participants explained that the association of alcohol with a famous personality would make the young children think that alcohol is prestigious and acceptable.

JSN, assisted by one of the research team members (WB), confirmed the availability of the alcoholic beverages and substances which were described by the participants. The ethanol content, alcohol by volume (ABV), of the bottled drinks mentioned by the participants were found to range from 2.6 to 40% ABV. The locally made drinks were sold from the production sites and other retail businesses, some of which were managed by children. The alcohol content of the locally made drinks was difficult to estimate but varied from strong spirits (waragi/ mandule – local names for the spirits) to less strong ones (malwa).

## Discussion

To our knowledge, this study represents the first attempt to explore the perspectives of children aged 6 to 13 years in Uganda regarding alcohol and other drug use by children. While researchers in high income countries (HICs) have reported on children’s understanding of alcohol [35], studies examining this topic in low-resource settings like Uganda have been scarce.

The main objective of this study was to gain insight into the perceptions held by children aged 6 to 13 years regarding AOD in the context of Uganda. Our findings revealed that children within the age range of 6 to 13, who are under adult care in Uganda, not only comprehend the processes associated with the production of homemade alcoholic beverages but also have access to various types of AOD and actively engage in their consumption. The participants’ narratives depict scenarios where children are exposed to alcohol consumption from an incredibly young age, including instances before language skills fully develop.

Moreover, our findings offer qualitative insights into the varying descriptions of the quantities of alcohol consumed by children, providing an understanding of this underexplored aspect of childhood experiences. The accounts from our study participants shed light on the diversity in alcohol consumption practices among children in our study area. Notably, children described a range of alcoholic beverages, from locally made brews taken in various containers to factory-made options available in packets and bottles. The descriptions also revealed varying consumption patterns, with some children reporting daily intake of multiple cups or bottles, particularly among the older age group (10–13 years). It is essential to note that the older children in our study acknowledged awareness of their peers consuming substantial quantities of alcohol, including beer and spirits, at times on a daily basis.

Importantly, our study underscores the discovery that children can access and consume alcohol from a diverse range of settings, including unexpected environments such as the classroom during instructional periods. Furthermore, our findings shed light on the complex interplay among child, family, school, and community factors, that contribute to childhood alcohol use. The discussion of these findings is organized in accordance with the social ecological model, originally developed Bronfenbrenner [4]. This model posts that child behaviours, including alcohol and drug use, are influenced by a complex interplay of factors at various levels of the child. Specifically, the child’s immediate surroundings, that is family and school (microsystem), as well as well as the interactions among these systems (mesosystems), and the broader social, political and economic conditions (exo-system), all play vital roles in determining child behavior. This model has been widely used to understand child mental health [36]. In light of this framework, our findings shed light on the interplay among child, family, school, and community factors including alcohol advertisement, which collectively contribute to childhood alcohol use. We present the child’s immediate environments and relationships (micro- and mesosystems) first and discuss the wider community and media (exo-system) thereafter.

### The child’s immediate environments and relationships

#### Parenting and role modelling

We found that families where alcohol use was prevalent were key contributors to the initiation and consumption of alcohol among children. Children discussed instances where parents consumed alcohol together with their children, or where children drank alcohol as a result of observing their parents’ drinking behaviour. This is consistent with previous research, such as studies using role plays scenarios, which found a link between childhood alcohol purchase and parental alcohol use [37, 38]. These findings suggest that children’s attitudes towards alcohol use may be influenced by the observation of their parents’ drinking habits.

Furthermore, participants in our study described instances where parents gave their children alcohol to drink, sometimes when they were hungry. These findings suggest that some parents in Uganda may not be aware of the potential risks associated with childhood alcohol use, which is sometimes considered nutritious, and that poverty and household food insecurity may be a driving factors [38]. Additionally, cultural norms and positive attitudes towards alcohol consumption among parents could also play a role. Previous research has found that some parents hold beliefs that promote childhood drinking, such as the idea that alcohol is not a drug or that sipping is not harmful to children [39, 40], which could contribute to parents giving their children alcohol [41]. More still, many participants discussed that children would easily access and drink alcohol from or outside the home, which indicates that their parents were not monitoring their activities, which made it easier for them to access and use AOD. However, research suggests that children whose parents establish and enforce clear family norms regarding alcohol use are more likely to delay initiation of drinking [42]. Moreover, Uganda has been identified as one of the countries in Africa with high rates of alcohol consumption [13, 43], indicating that many children may be living in families where alcohol use is common. Overall, these conclusions suggest that parents play a crucial role in shaping their children’s attitudes towards alcohol and other drug use. Therefore, it is essential to educate parents about the risks associated with early alcohol and drug consumption and encourage them to model responsible drinking behaviours.

#### Home brewing and factory brands

The participants in this study reported that childhood initiation of alcohol and other drug (AOD) use was often influenced by their parents, siblings, or friends who consumed alcohol. They demonstrated detailed knowledge of various types of alcohol and other drugs and their effects. Many participants were familiar with the manufacturing processes for homemade alcoholic beverages as well as the packaging for factory-made types. This exposure to alcohol and other substances could be attributed to their daily interactions with adults and peers who consumed alcohol. In other studies, children of brewers in the Karamoja region of Uganda were found to be involved in the brewing process, leading to early initiation [38]. Adults in Mbale similarly reported that children were exposed to alcohol and other substances at a young age because their parents brewed at home [2]. Earlier studies in Uganda indicated that locally available alcoholic beverages were brewed at home, and women were often involved in production to pay for their children’s school fees [44]. These studies suggest a link between childhood alcohol use and the selling of home-brewed beverages.

#### Effects and attitudes towards alcohol and other drug use

The children in this study demonstrated a clear understanding of the short- and long-term consequences of AOD use. They described physical effects such as a bad smell, getting drunk, feeling sleepy, dizzy, forgetful, having headaches, being prone to accidents, and even death. Children who used marijuana were described as having red eyes, black teeth, and a disturbed mental status. The participants also discussed the negative behavioural consequences of AOD use, such as disrespect for parents and teachers, fighting, stealing, and rape. They acknowledged that these behaviours would lead to problems at school, dropping out, or committing crimes that would result in punishment or arrest. The participants also recognized that their behaviour would affect other children who did not use AODs, causing them to become fearful of being raped or stoned. These findings are consistent with previous research that has shown that children’s knowledge of the negative effects of AOD use and the behaviours of users could delay initiation [45].

#### Childhood adversities

This study identified several reasons for childhood AOD use that were related to stresses from adverse childhood experiences and childhood adversities. For example, participants discussed how parental separation due to family conflict or the death of a mother, resulting maltreatment (especially from stepmothers), and physical punishments by teachers contributed to childhood AOD use. Additionally, parents who drank alcohol often did so because of hardships, such as when they gave their babies alcohol to help them sleep. This is consistent with previous research that has found that families undergoing hardships such as poverty and the lack of basic needs often exhibit maladaptive behaviours such as drinking alcohol or using other drugs [46]. Childhood adversities have also been shown to be associated with early and problematic AOD use [47]. While limited literature exists on childhood alcohol consumption in LMICs, some studies in Uganda have described adversities such as hunger and problematic family situations contributing to childhood alcohol use [2, 38]. These findings underscore the importance of understanding the impact of childhood adversities on AOD use in LMICs.

### Wider community and media

Children in this study reported that the branding and advertising of factory-made alcoholic beverages made them look “cool” and attractive. They were able to recognize and name several specific brands, such as “Empire.“ This finding is consistent with previous research that has found media content and labelling to be strongly associated with the initiation of alcohol consumption and the use of other drugs [48, 49]. Furthermore, children in this study reported being able to purchase alcohol directly from bars, shops, and supermarkets, as well as from local manufacturers and brewers, at prices as low as 200 to 1000 Uganda shillings or even for free. These findings highlight the need for increased regulation of alcohol marketing and sales to children in LMICs.

Although alcohol packaged in sachets was prohibited in Uganda, much of it was sold in small bottles which were easily accessible by school-age children. The ease with which children could get alcohol even outside home has not been fully described in this context previously. The participants discussed that children sometimes had to provide cheap labour, steal or lie in order to get alcohol. The fact that school-age children in Uganda are able to access alcohol through such means as providing cheap labour, stealing, or lying is concerning and highlights the need for increased efforts to prevent underage drinking. [1, 50].

### Implications for clinical practice, policy and programs

The study’s results have far-reaching implications for clinical practice, policy, and program development. Firstly, it is crucial to acknowledge the potential social and public health risk of children’s alcohol use across all sectors. The Uganda health system needs to identify at-risk children for timely prevention and care, involving primary health workers, paediatricians, and other sectors where children are present.

Additionally, this study reveals gaps in the implementation of Uganda’s alcohol policy and legislation at various levels, including homes, schools, and communities. The beverages consumed by children had alcohol content as high as 40% ABV, yet most containers had a warning against selling alcohol to persons below the age of 18 years. Therefore, there is an urgent need to review the existing Ugandan alcohol policies and regulations, improve their awareness and implementation at all levels, and consider children’s protection.

It is important for policymakers and public health officials to consider strategies to limit children’s access to alcohol, such as stronger enforcement of existing laws prohibiting the sale of alcohol to minors, as well as public awareness campaigns aimed at parents, educators, and other caregivers on the risks of underage drinking.

### Strengths and limitations

This is the first study to capture the perspectives of children aged 6 to 13 years regarding alcohol and other drug use in Uganda. The participants were children themselves and had a diverse representation of views from different age groups, sex, and in or out of school categories that enabled us to draw conclusions on the child perspectives regarding childhood alcohol and drug use. However, the FGDs were conducted within Mbale city and represent the views of those children who participated in the study and may not be generalizable to all school age children in Uganda. In addition, due to the qualitative nature of the study and the absence of quantitative data collection, we were unable to calculate the mean age or provide specific percentages for various characteristics. Despite these limitations we believe that the findings from this study could inform further policy as well as designing of research, prevention and intervention programs.

## Conclusions

This study found significant insights regarding the perspectives of children aged 6 to 13 years under adult care in Mbale district, eastern Uganda, who possess knowledge of and easy access to alcohol and other drugs. The identified contributing factors encompass a range of influences, including availability, accessibility, advertising and promotion of alcoholic beverages, lack of parental awareness and supervision, sibling and peer dynamics, adverse childhood experiences, socio-economic factors, and cultural norms. These findings underscore the urgency of addressing childhood alcohol and drug use to safeguard the well-being of children in the region.

### Recommendations

We advocate for the implementation of targeted awareness and education campaigns aimed at parents and communities, with the goal of safeguarding children. Furthermore, we recommend the training of health professionals and teachers to effectively identify at-risk children and provide timely prevention and care services. To address the knowledge gap, we suggest conducting epidemiological studies to determine the extent of alcohol use by children. Implementation research involving children, parents, educators, healthcare professionals, and policymakers should be conducted to capture the diverse perspectives and collaboratively develop evidence based programs and interventions aimed at mitigating children’s alcohol and drug use. We also propose in-depth policy analyses to assess the alignment between existing alcohol and drug policies and the emerging understanding of childhood alcohol use, identifying potential gaps and opportunities for revision and implementation.

Overall, the findings of this study may be used to inform collaborative efforts and partnerships among researchers, policymakers, practitioners and other stakeholders to address childhood alcohol use in Uganda and other low resource settings.

## Data Availability

Data is available upon reasonable request from the corresponding author. Both Makerere University School of Public Health and the Center for International Health, University of Bergen have shared intellectual property rights to the data.

## Declarations

### Authors’ information

The first author (JSN) is a Ugandan female psychiatrist with special interest and training in child and adolescent psychiatry and mental health, which she has practiced for the past 10 years. She holds a Bachelor of Medicine and a Master of Medicine in Psychiatry.

RN is a female social worker with a master’s degree in social work with extensive experience in qualitative research. WB is a male social worker with a master’s degree in social work with experience in qualitative data collection. Both RN and WB were from Mbale district and knew the local languages as well as English. All the co-authors have experience with qualitative research.

## List of abbreviations

AOD: Alcohol and other Drugs
FGD: Focus group discussion
HIC: High Income Country
HIV/AIDS: Human Immune Deficiency Virus/ Acquired Immuno deficiency Syndrome
LMICs: Low-and Middle Income Countries
SOMHDREC: School of Medicine Higher Degrees Research Ethics Committee
UBOS: Uganda National Bureau of Statistics
UNCST: Uganda National Council for Science and Technology
WHO: World Health Organization
